# Correction

**Published:** 1885-11

**Authors:** 


CORRECTION.—The Scalers used by Dr. A. W. Harlan in his clinic on Pyorrhea Alveolaris, at the Minneapolis meeting, were not those of Dr. Allport, as incorrectly stated in the report of proceedings (page 538), but those of his own invention, as described on page 556 of the October issue. The inaccuracy in the report is regretted.—" MRS. M. W. J."

